# Emergence and characterization of a ST852 *Klebsiella quasipneumoniae* clinical isolate coharboring *bla*_NDM-1_ and *bla*_KPC-2_ in China

**DOI:** 10.3389/fcimb.2025.1564277

**Published:** 2025-05-09

**Authors:** Chongmei Tian, Yaping Zhao, Su Dong, Zhixin You, Jingbai Chen, Hongfeng Xu, Yuejuan Fang, Yapei Zhang

**Affiliations:** ^1^ Department of Pharmacy, Shaoxing Hospital of Traditional Chinese Medicine Affiliated to Zhejiang Chinese Medical University, Shaoxing, Zhejiang, China; ^2^ Department of Clinical Laboratory, Shaoxing Hospital of Traditional Chinese Medicine Affiliated to Zhejiang Chinese Medical University, Shaoxing, Zhejiang, China; ^3^ Department of Respiratory Medicine, Shaoxing Hospital of Traditional Chinese Medicine Affiliated to Zhejiang Chinese Medical University, Shaoxing, Zhejiang, China; ^4^ Pharmacy Department of Chinese Medicine, Shaoxing Hospital of Traditional Chinese Medicine Affiliated to Zhejiang Chinese Medical University, Shaoxing, Zhejiang, China; ^5^ Department of Pharmacy, Quzhou Maternal and Child Health Care Hospital, Quzhou, China; ^6^ Department of Clinical Laboratory, Zhejiang Hospital, Hangzhou, Zhejiang, China

**Keywords:** *K. quasipneumoniae*, WGS, ST852, NDM-1, KPC-2, plasmid structure

## Abstract

**Objectives:**

To characterize a rare ST852 *Klebsiella quasipneumoniae* strain co-producing NDM-1 and KPC-2 isolated from a clinical patient.

**Methods:**

Minimum inhibitory concentrations (MICs) were measured using a VITEK 2 compact system and broth microdilution. Conjugation experiments were conducted using film matings. Whole genome sequencing (WGS) was performed using Illumina and Nanopore platforms. Antimicrobial resistance determinants were identified using the ABRicate program in the ResFinder database. Insertion sequences (ISs) were identified using ISFinder. Bacterial virulence factors were identified using a virulence factor database (VFDB). Genome function annotation and classification were further analyzed using the Kyoto Encyclopedia of Genes and Genomes (KEGG) and Cluster of Orthologous Groups (COG) databases. Capsular polysaccharides (KL) and lipooligosaccharides (OCL) were tested using Kleborate with the *Kaptive*. Multilocus sequence typing (MLST) and replicon types were identified using the Center for Genomic Epidemiology website. Prophage region analysis was performed using PHASTEST software. Conjugation-related elements were predicted using *oriT*finder. The plasmid structure was visualized using Circos and similar plasmids in the public database were tracked using BacWGSTdb. A global phylogeny for the ST852 *K. quasipneumoniae* isolates was further performed.

**Results:**

*K. quasipneumoniae* KPSY isolate was identified as ST852, with KL18 and O3/O3a. It has an extensive drug-resistant (XDR) profile. WGS analysis revealed that it contained one circular chromosome and three plasmids. The results of the COG and KEGG functional classifications showed that most of the functions were associated with metabolism. pKPSY-2 is a 239,226-bp IncU plasmid carrying the carbapenem resistance gene *bla*_NDM-1_. pKPSY-3 is a smaller plasmid belonging to the IncN-type conjugative plasmid with *bla*_KPC-2_. Importantly, *oriT* sequence, the T4SS region, T4CP, and relaxase were identified. Tracking of the *bla*_KPC-2_ plasmids showed they were identified in different species in different countries, including *E. coli*, *Leclercia* sp., *Pantoea* sp., and *E. hormaechei*. Global analysis data showed 13 ST852 strains were mainly isolated from China (84.62%, 11/13), and the remaining isolates were collected from Switzerland.

**Conclusions:**

This is the first study to identify an ST852 NDM-1-KPC-2 coproducing *K. quasipneumoniae* clinical isolate. Surveillance is warranted, and early detection of this high-risk clone in the clinic is recommended to avoid its extensive spread.

## Introduction

Carbapenem-resistant Enterobacteriaceae (CRE) are known to cause a variety of important nosocomial infections associated with high mortality rates and are a global public health threat to patients in hospital settings (Hala et al., 2019). The treatment of CRE is considered a tremendous challenge by the US Centers for Disease Control and Prevention (CDC) (Gu et al., 2018). A majority of CRE-associated genes have been identified, including the New Delhi Metallo-beta-lactamases (NDMs) and *Klebsiella pneumoniae* carbapenemases (KPCs) (Krapp et al., 2018; Bar-Yoseph et al., 2019).

*Klebsiella pneumoniae* (KP) was initially classified into three closely related phylogroups. Based on genome sequencing, they are classified into three distinct species: *K. pneumoniae*, *K. quasipneumoniae*, and *K. variicola* (Holt et al., 2015). Their distinction was possible through the comparison of core genomes and not through conventional multi-locus sequence typing (MLST) and capsule genotyping (Long et al., 2017). Importantly, all three species cause human infections (Holt et al., 2015).

Because of the significant overlap in their biochemical profiles, phenotypic testing using traditional microbiological assays is incapable of accurately differentiating *K. pneumoniae*, *K. quasipneumoniae*, and *K. variicola* (Sakai and Maesaki, 2022). Among these, *K. quasipneumoniae* was reported as an emerging pathogen in 2004 that could cause bloodstream infections (BSIs) in healthy individuals (Dong et al., 2023). Comparative genomics is crucial for understanding the relationships and distinctions between these organisms, facilitating accurate and timely diagnosis and clinical management (Long et al., 2017).

Here, a rare ST852 *K. quasipneumoniae* clinical isolate co-harboring *bla*_NDM-1_ and *bla*_KPC-2_ was isolated from wound secretion in 2022 in China and its complete genetic characteristics were studied. To the best of our knowledge, this is the first study to identify an ST852 NDM-1-KPC-2 coproducing *K. quasipneumoniae* clinical isolate. Importantly, the combination of Illumina and MinION whole-genome sequencing (WGS) approaches provides complete insight into the genomic structural features of the important resistance plasmids found in rare *K. quasipneumoniae* isolates of clinical origin.

## Materials and methods

### Bacterial isolation and identification

*K. quasipneumoniae* KPSY isolate was collected from a teaching hospital in China during routine diagnostic analysis of wound secretion of a 42-year-old male patient from the intensive care unit (ICU) in 2022 in Zhejiang province, China. Isolate identification at the species level was conducted using matrix-assisted laser desorption ionization time-of-flight mass spectrometry (MALDI-TOF MS; Bruker Daltonics GmbH, Bremen, Germany) and confirmed using the fIDBAC database (http://fbac.dmicrobe.cn/home/).

### Antimicrobial susceptibility testing

Minimum inhibitory concentrations (MICs) were measured using the VITEK 2 compact system: amikacin, aztreonam, ertapenem, imipenem, meropenem, levofloxacin, ciprofloxacin, ceftazidime, ceftriaxone, cefazolin, cefepime, cefoxitin, gentamicin, tobramycin, and piperacillin. The MIC of colistin was determined using the broth microdilution method. Experimental data were interpreted according to the recommendations of the Clinical and Laboratory Standards Institute (CLSI) 2021 guidelines.

### Hypermucoviscous phenotype determination

The string test was performed based on our previous study (Tian et al., 2022). In brief, strain was cultured on a sheep blood agar plate and then performed overnight culture at 37°C followed by streaking an inoculation loop through a colony next day. If a string of > 5 mm was positive, the strain was considered hypermucoviscous.

### Plasmids conjugation assays via film mating

To determine the transferability of *bla*_KPC-2_ or *bla*_NDM-1_ positive plasmids, conjugation experiments using *E. coli* EC600 (rifampin-resistant) as the recipient strain were conducted using the film-mating method (Yang et al., 2021; Tian et al., 2022). Transconjugants were screened on Mueller–Hinton agar plates containing rifampin (150 μg/mL) and meropenem (2 μg/mL). The identities of the putative transconjugants were confirmed using PCR using specific primers and MALDI-TOF MS. Experiments were independently performed three times.

### Short-read and long-read whole genome sequencing

#### Short-read illumina sequencing

Genomic DNA was extracted using a Qiagen Mini kit (Qiagen, Hilden, Germany), in accordance with the manufacturer’s recommendations. The quality and quantity of the DNA were assessed using a NanoDrop 2000 (Thermo Scientific, USA) and a Qubit 4.0 fluorometer (Invitrogen, USA). Libraries were prepared using the TruePrepTM DNA Library Prep Kit V2 (Vazyme). Individual libraries were assessed with the QIAxcel Advanced Automatic Nucleic Acid Analyzer using a high-resolution gel cartridge (Qiagen, Germany) and then quantified using qPCR using the KAPA SYBR FAST qPCR Kit Kapa KK4610 (KAPA Biosystems, Wilmington, MA, U.S.A.). Paired-end sequencing was performed using an Illumina HiSeq X Ten platform (Illumina, San Diego, CA, USA).

#### Long-read Oxford Nanopore sequencing

Long-read sequencing was performed for the strain. DNA was extracted using the Gentra^®^ Puregene^®^ Yeast/Bact. kit (Qiagen, Germany), according to the manufacturer’s protocol. Oxford Nanopore sequencing libraries were prepared using the SQU-LSK109 Ligation Sequencing Kit in conjunction with the PCR-Free ONT EXP-NBD104 Native Barcode Expansion Kit (Oxford Nanopore Technologies, UK). DNA was processed without the optional shearing steps to select long reads. After quantification of the individual libraries using Qubit and normalization of library concentrations, the libraries were sequenced using the GridION X5 platform (Oxford Nanopore Technologies, UK).

### Bioinformatic analyses for the genomes

Hybrid assembly of the short and long reads of Illumina and MinION was constructed using Unicycler v0.4.8 (Wick et al., 2017). Genome annotation was performed using the National Center for Biotechnology Information (NCBI) Prokaryotic Genome Annotation Pipeline (PGAP) (http://www.ncbi.nlm.nih.gov/genome/annotation_prok/ ) (Tatusova et al., 2016) and Prokka (Seemann, 2014). Genome sequence function annotation and classification were further analyzed using the Kyoto Encclopedia of Genes and Genomes (KEGG) and Cluster of Orthologous Groups (COG) databases. Antimicrobial resistance genes were identified using ABRicate program (https://github.com/tseemann/abricate) using the ResFinder database and the Center for Genomic Epidemiology website (https://genomicepidemiology.org/). Virulence factors were identified using the virulence factor database (VFDB, http://www.mgc.ac.cn/VFs/) (Liu et al., 2022). Capsular polysaccharide (K locus) and lipooligosaccharide (OC locus) were analyzed using Kleborate with the command line of kleborate, ASSEMBLIES-k (Lam et al., 2021, Lam et al., 2022). Multilocus sequence typing (MLST) and replicon types were identified using the Center for Genomic Epidemiology website (https://genomicepidemiology.org/). Prophage region analysis of the genome was conducted with PHASTEST (PHAge Search Tool with the Enhanced Sequence Translation) web server (https://phastest.ca/).

For the analysis of *bla*_KPC-2_- and *bla*_NDM-1_-positive plasmids, conjugation transfer elements, including the origin site of DNA transfer (*oriT*), type IV secretion system (T4SS) region, type IV coupling protein (T4CP), and relaxase, were predicted using *oriT*finder with default parameter settings (Li et al., 2018). The insertion sequences (ISs) were identified using ISfinder (Siguier et al., 2006). Plasmid structure was visualized using DNAplotter (Carver et al., 2009) and Circos (Krzywinski et al., 2009). Similar plasmids in public databases were tracked using the BacWGSTdb (http://bacdb.org/BacWGSTdb) (Ruan et al., 2020; Feng et al., 2021). Plasmids comparisons were conducted using Proksee (https://proksee.ca/projects/new). Default parameters were used for all software packages.

### Global analyses for ST852 *K. quasipneumoniae* isolates

To construct a global phylogeny for the ST852 *K. quasipneumoniae* isolates, we downloaded 2,639 genome sequences from the National Center for Biotechnology Information (NCBI) database (search conducted on 1.4.2025). All genome sequences were analyzed by mlst v2.19.0 command and ABRicate v1.0.1 (Liu et al., 2021). Quality control was performed using FastANI v1.33 and QUAST v5.0.2 (Gurevich et al., 2013). As a result, the obtained ST852 genomes were further sent to the further epidemiology and genetic relationship analysis. Snippy v4.4.5 (https://github.com/tseemann/snippy) was used to generate a core genome alignment. Phylogenies were constructed using FastTree (Price et al., 2009). The resulting tree was annotated and visualized using iTOL v7 (https://itol.embl.de/).

## Results

### MICs and antimicrobial resistance genes profile

The antimicrobial susceptibility testing data revealed *K. quasipneumoniae* KPSY clinical isolate possessed an extensively drug-resistant (XDR) profile, with the imipenem, meropenem, and ertapenem MICs > 8 μg/mL (**Table 1**). Furthermore, *K. quasipneumoniae* KPSY strain was resistant to many antimicrobial agents, including amikacin, aztreonam, levofloxacin, ciprofloxacin, ceftazidime, ceftriaxone, cefazolin, cefepime, cefoxitin, gentamicin, tobramycin, and piperacillin (**Table 1**). However, it was still susceptible to colistin (< 0.125 μg/mL).

**Table 1:** Minimal inhibitory concentrations (MICs) to different antimicrobial agents of *K. quasipneumoniae* KPSY.

Antimicrobials	MIC (μg/mL)	Susceptibility
Amikacin	> 32	R
Aztreonam	> 16	R
Ceftazidime	> 16	R
Ciprofloxacin	> 2	R
Levofloxacin	> 4	R
Ceftriaxone	> 32	R
Cefazolin	> 16	R
Cefepime	> 16	R
Cefoxitin	> 16	R
Gentamicin	> 8	R
Tobramycin	> 8	R
Piperacillin	> 4	R
Ertapenem	> 8	R
Imipenem	> 8	R
Meropenem	> 8	R
Colistin	< 0.125	S

S, susceptible; R, resistance.

Analysis of the genome of *K. quasipneumoniae* KPSY clinical isolate revealed that, in addition to co-harboring chromosomal *bla*_OKP-B-2_, *fosA*, *oqxA* and *oqxB*, a series of genes conferring resistance to β-lactams (*bla*_NDM-1_, *bla*_KPC-2_), aminoglycosides (*aac(6’)-Ib3*, *aadA16*, *aadA1*), sulfonamides (*sul1*), trimethoprim/sulfamethoxazole (*dfrA27*), quinolones (*qnrS1*), rifampicin (*ARR-3*), chloramphenicol (*catB3*), phenicol (*cmlA1*), fosfomycin (*fosE*) and bleomycin (*ble-MB*L) (**Table 2**).

**Table 2:** Molecular characterization of genome of the KPSY clinical strain.

Genome	Size (bp)	Replicon	GC content	Resistance genes	Accession number
Chromosome	5,185,420	ND	ND	*bla*_OKP-B-2_, *fosA*, *oqxA*, *oqxB*	SAMN45957920
pKPSY-1	342,682	IncFIB(K)	50%	ND	SAMN45957920
pKPSY-2	239,226	IncU	47%	*aac(6’)-Ib3*, *aadA16*, *bla*_NDM-1_, *ble-MBL*, *catB3*, *ARR-3*, *dfrA27*, *qnrS1*, *sul1*	SAMN45957920
pKPSY-3	58,981	IncN	52%	*bla*_KPC-2_, *aadA1*, *cmlA1*, *fosE*, *aac(6’)-Ib3*, *sul1*	SAMN45957920

ND, Not detected.

### Virulence factors in *K. quasipneumoniae* KPSY isolate

There are many virulence factors in the *K. quasipneumoniae* KPSY isolate, including iron-enterobactin transporter-related protein genes (*fepABCDG*), siderophore esterase (*iroE*), type 1 fimbrial (*fimABCDEFGHI*), type 3 fimbriae (*mrkABCDFHIJ*), and many others (*tli1*, *vasE/tssK*, *vasE/tssK*, *vasE/tssK*, *clpV/tssH*, *dotU/tssL* and *vipB/tssC*). However, the result of Kleborate showed that the virulence score is 0. Consistent with the Kleborate data, no genes encoding yersiniabactin, colibactin, aerobactin, salmochelin, RmpADC and RmpA2 were found in our strain. Moreover, the string test experiment is negative.

### Multilocus sequence typing, KL and OCL

Based on the *K. pneumoniae* MLST scheme, *K. quasipneumoniae* KPSY isolates were typed as ST852 (*gapA*-18, *infB*-22, *mdh*-18, *pgi*-22, *phoE*-139, *rpoB*-69, *tonB*-179). Kleborate showed that *K. quasipneumoniae* KPSY strain was KL18, with 96.32% nucleotide identity and 99.93% coverage. The O locus was subjected to O3/O3a, matching 98.52% and 99.77% nucleotide identity.

### Chromosome and plasmids characterization of the KPSY strain

The hybrid assembly showed that *K. quasipneumoniae* KPSY strain had a 5,185,420-bp size circular chromosome (**Table 2**). The *bla*_OKP-B-2_ gene was identified on this chromosome. Moreover, no amino acid mutations in GyrA (S83F) or ParC (S80I), which confer resistance to fluoroquinolones, were found.

Three plasmids were identified in our *K. quasipneumoniae* KPSY strain, namely pKPSY-1 to pKPSY-3, with sizes between 58,981-bp to 342,682-bp and GC contents ranging from 47% to 52% (**Table 2**). pKPSY-1 is an IncFIB(K)-type plasmid and no resistance was detected. The pKPSY-2 and pKPSY-3 plasmids have different multidrug resistance (MDR) plasmids harboring various important resistance genes. pKPSY-2 is an IncU plasmid carrying the carbapenem-resistance gene *bla*_NDM-1_. pKPSY-3 is the smaller plasmid, which belonged to IncN-typed plasmid. It possesses six antimicrobial resistance (AMR) genes: *bla*_KPC-2_, *aadA1*, *cmlA1*, *fosE*, *aac(6’)-Ib3*, and *sul1*.

### Function classification of COG and KEGG

**Figure 1:**
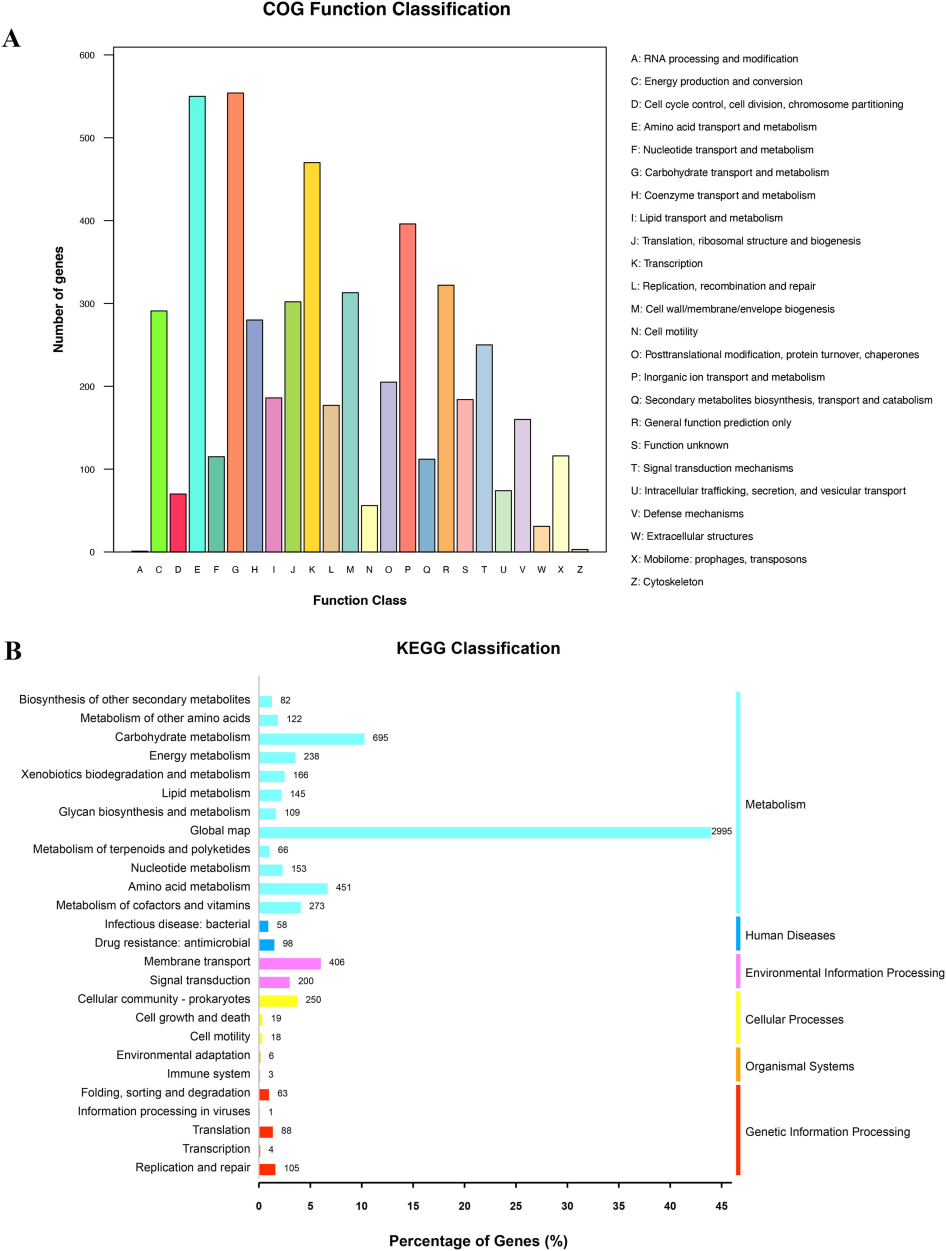
Counts of subsystem based on COG and KEGG annotation. **(A)** COG function annotation and classification. **(B)** KEGG classification.

The results of the COG and KEGG functional classification showed that the majority of the functions were associated with metabolism, including lipid transport and metabolism, amino acid metabolism, and nucleotide metabolism (**Figures 1A, B**). In addition, the functions of cell motility, replication, and repair were classified using both the COG and KEGG databases (**Figures 1A, B**).

### Prophage regions in the genome

Prophage regions were predicted using the PHASTEST tool, and the results showed one intact, one questionable, and four incomplete regions on the chromosome of the *K. quasipneumoniae* KPSY strain (**Table 3**). Region 1 in the chromosome was in length of 37.9 kb. It was predicted to be intact because of a score > 90 and a protein number of 43. In addition, Region 3 was classified as questionable with a score of 80. Among the three plasmids, only one intact prophage region was found in pKPSY-1 (protein number: 29). The majority of the prophage regions reported in the plasmids were incomplete owing to their low scores (**Table 3**).

**Table 3:** Prophages regions in the genome of *K. quasipneumoniae* KPSY.

Genome	Region	Length	Score	Protein number	Region position
Chromosome	1	37.9Kb	Intact (107)	43	650277-688267
Chromosome	2	23.1Kb	Incomplete (60)	14	1222538-1245733
Chromosome	3	45.9Kb	Questionable (80)	23	2661045-2706973
Chromosome	4	15.4Kb	Incomplete (30)	22	2696708-2712112
Chromosome	5	20.2Kb	Incomplete (40)	25	3377721-3397964
Chromosome	6	27.4Kb	Incomplete (60)	25	3398664-3426131
pKPSY-1	1	5.3Kb	Incomplete (40)	7	41326-46722
pKPSY-1	2	8Kb	Questionable (70)	13	143611-151624
pKPSY-1	3	19.8Kb	Incomplete (60)	18	157460-177347
pKPSY-1	4	22.8Kb	Intact (100)	29	204005-226875
pKPSY-1	5	6.2Kb	Incomplete (40)	11	335738-341943
pKPSY-2	1	11.6Kb	Incomplete (20)	27	93262-104893
pKPSY-2	2	11.2Kb	Incomplete (10)	21	117887-129147
pKPSY-2	3	11.2Kb	Incomplete (60)	17	196731-207962
pKPSY-3	1	12.8Kb	Incomplete (40)	17	1-12858
pKPSY-3	2	13.5Kb	Incomplete (50)	14	30167-43716

### Genetic features of IncU *bla*_NDM-1_-positive plamid pKPSY-2

**Figure 2:**
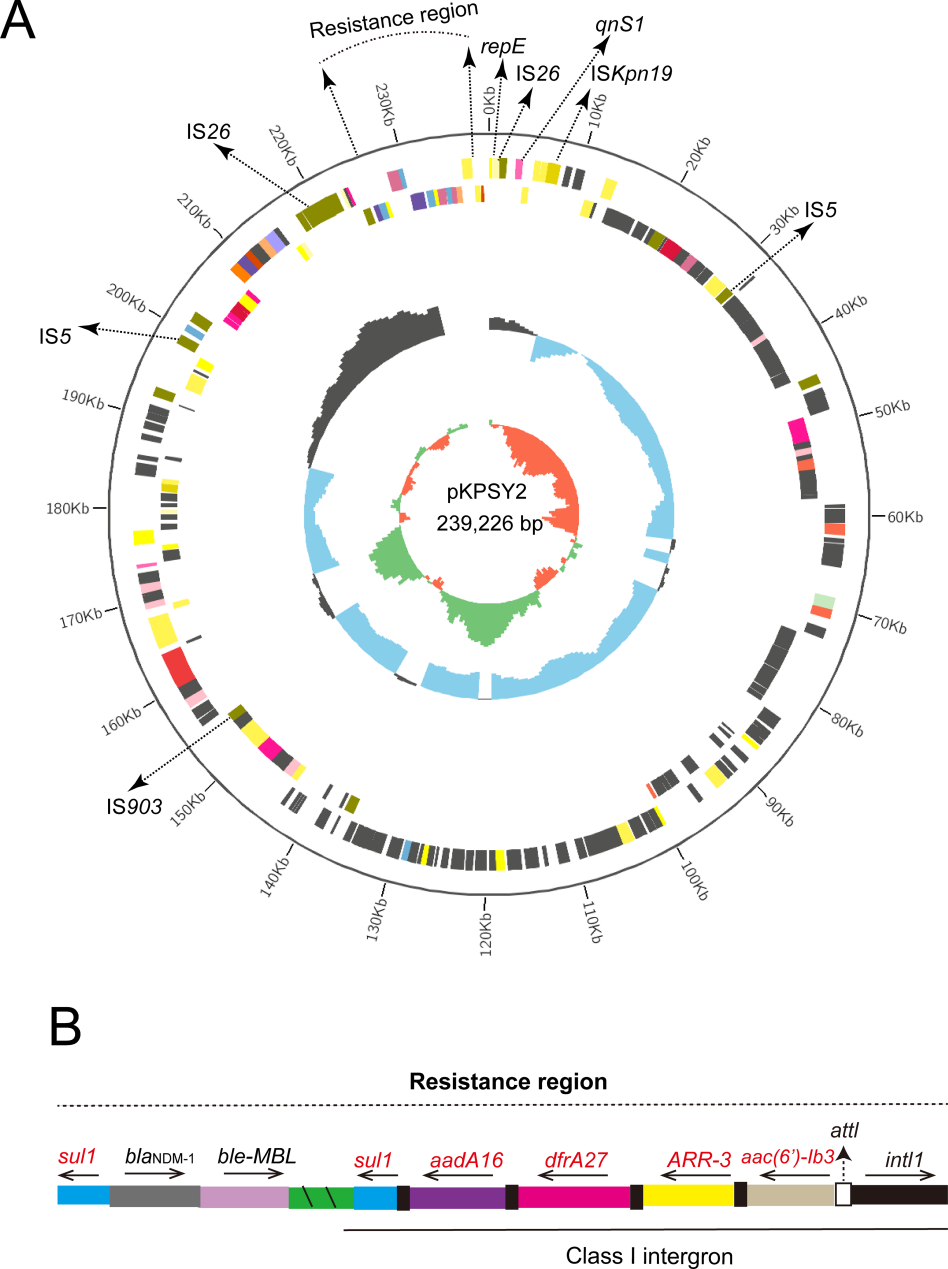
Circular map of pKPSY-2 and the genetic structure of the *bla*_NDM-1_. **(A)** Circular map of pKPSY-2 plasmid. **(B)** Structure of the resistance region containing *bla*_NDM-1_ gene and class I intergron.

*K. quasipneumoniae* KPSY clinical isolate harbored a 239,226-bp plasmid, pKPSY-2, with an IncU replicon. The structure of the plasmid is shown in **Figure 2A**. Downstream of *bla*_NDM-1_ is the bleomycin resistance-related gene *ble-MBL*. However, IS*Aba125* was not found upstream of *bla*_NDM-1_. Moreover, one class 1 integron with the *intI1*-*aac(6’)-Ib3-ARR-3-dfrA27-aadA16-sul1* genetic array was identified in the pKPSY-2 plasmid **Figure 2B**. In addition, many mobile genetic elements (MGEs), including IS*26*, IS*5*, IS*EC33*, and IS*Kpn19*, were detected. Two copies of IS*26* were detected in the pKPSY-2 plasmid. One copy of IS*26* was flanked by an 8-bp direct repeat (DR) sequence (ACTTCGAG and GAAGCAGA). Another copy of IS*26* was flanked by different 8-bp DR sequences (CGCTGGAC and TTTTCGAG). Additionally, the results of *oriT*finder showed that no *oriT*, T4SS region, T4CP, or relaxase were found in this plasmid. Mating assays were performed to analyze the transfer ability of *bla*_NDM-1_; however, the results showed *bla*_NDM-1_ failed to transfer to the recipient strain.

### Characterization of the KPC-2-producing plasmid pKPSY-3

**Figure 3:**
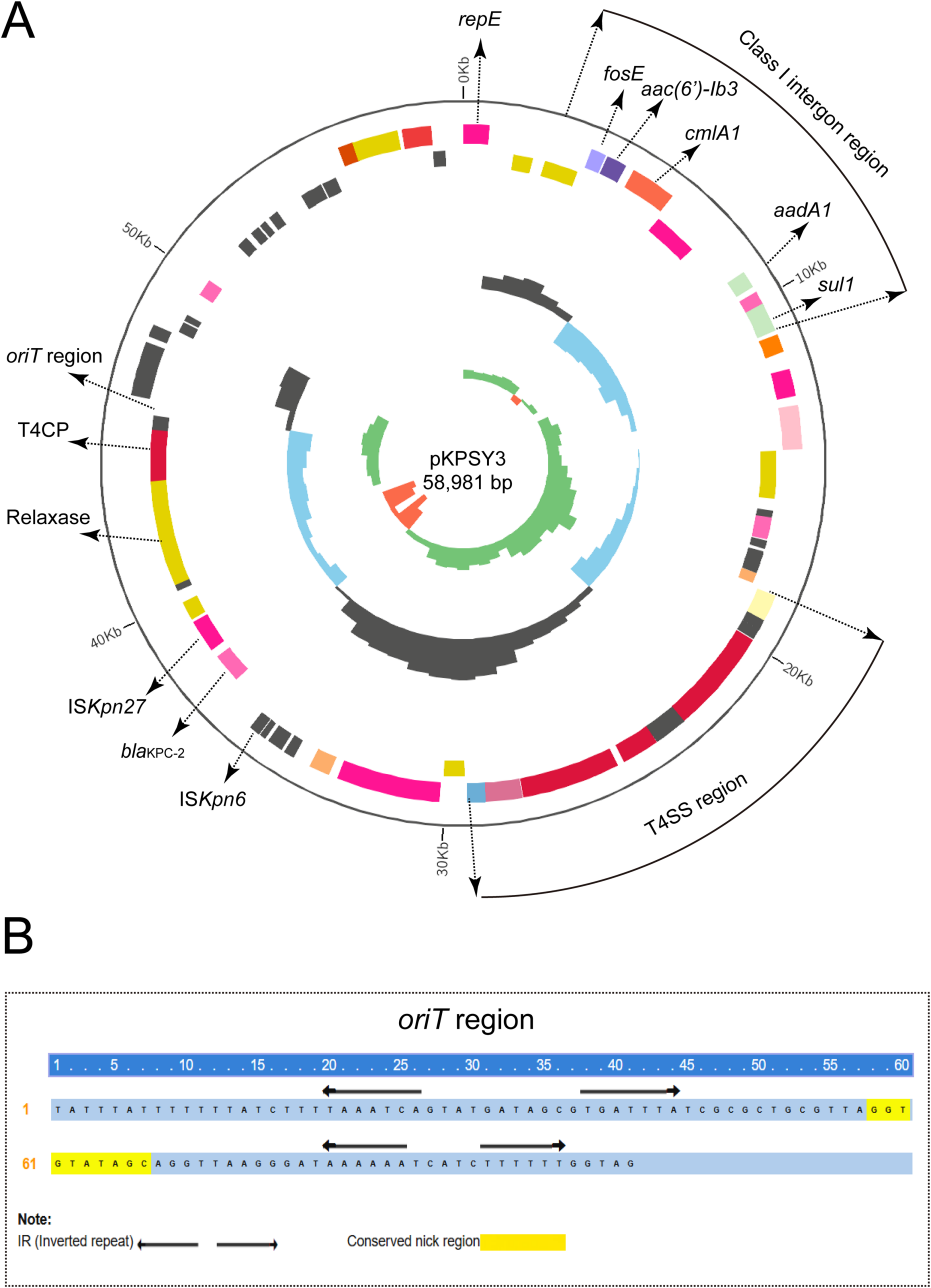
Circular map of pKPSY-3 and the genetic structure of the *bla*_KPC-2_. **(A)** Circular map of pKPSY-3 plasmid. The T4SS region and class I intergron were labelled. The *oriT*, T4CP and relaxase were also shown. **(B)** Sequence of the *oriT* region. IR and the conserved nick region were shown.

Based on bioinformatic analysis, *bla*_KPC-2_ carbapenem gene was located in a 58,981-bp plasmid, designated pKPSY-3, with an IncN-type replicon. pKPSY-3 had an average GC content of 52% and comprised different regions, including the T4SS and class 1 integron regions (**Figure 3A**). The resistance genes array of *intI1*-*fosE*-*aac(6’)-Ib3-cmlA1-aadA1-sul1* was identified in the pKPSY-3 plasmid (**Figure 3A**). The genetic context of *bla*_KPC-2_ is IS*Kpn27*-*bla*_KPC-2_-IS*Kpn6*. Importantly, *oriT* sequence, the T4SS region, T4CP, and relaxase were identified in the plasmid. The inverted repeat (IR) and conserved nick regions of the *oriT* sequence are shown in **Figure 3B**. Conjugation experiments demonstrated that it could be transferred to the recipient strain.

### Track of similar plasmids in public database

**Figure 4:**
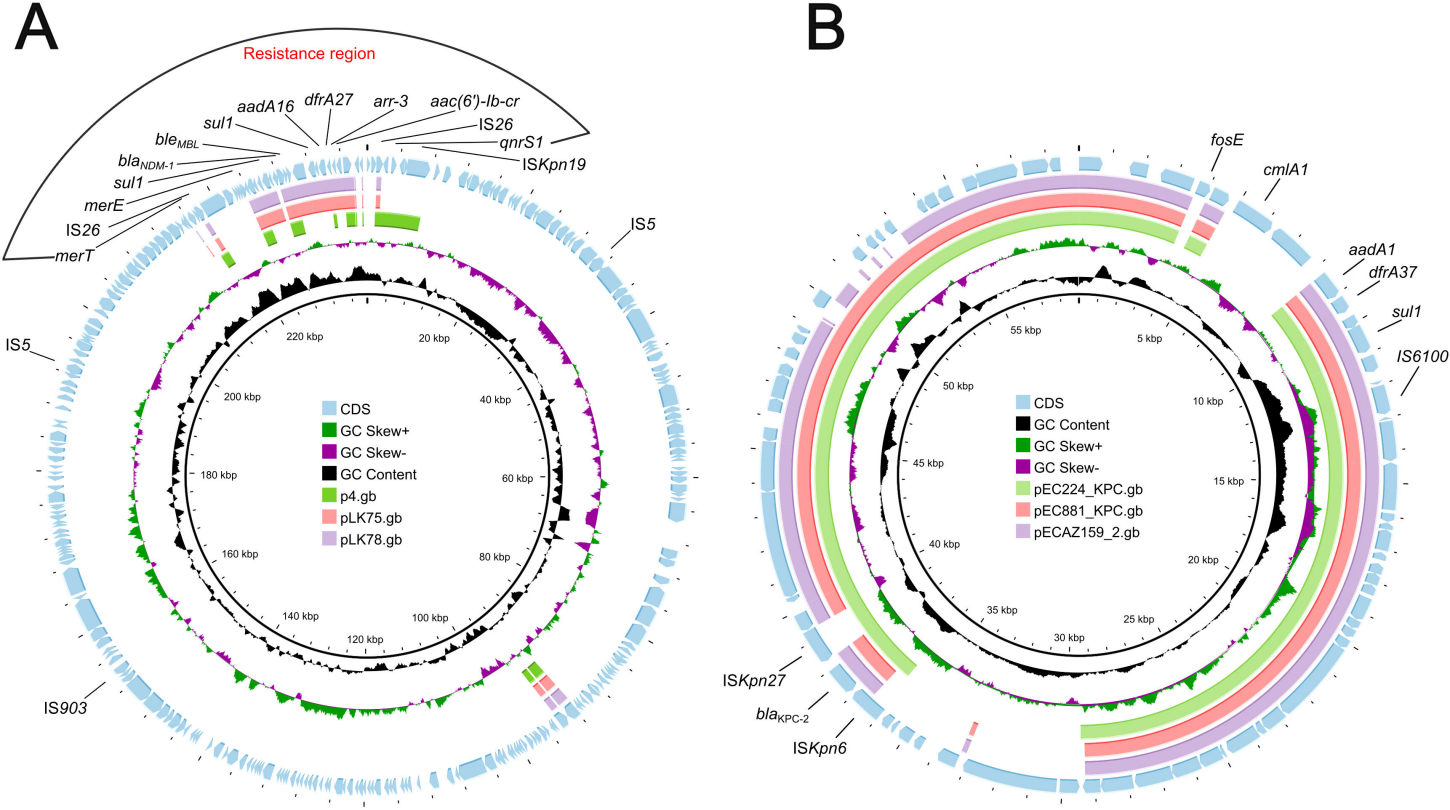
Plasmids comparison of *bla*_NDM-1_-harboring and *bla*_KPC-2_-positive plasmids. **(A)** *bla*_NDM-1_-harboring plasmids comparison using Proksee. pKPSY-2 was used as reference plasmid. Plasmids p4, pLK75 and pLK78 were compared with pKPSY-2. **(B)** *bla*_KPC-2_-positive plasmids comparison. pKPSY-3 was used as reference plasmid. Plasmids pEC224_KPC, pEC881_KPC and pECA159_2 were compared with pKPSY-3.

Comparative genomic analysis was performed using the BacWGSTdb server to analyze similar plasmids from different countries. Information on similar plasmids is shown in **Table 4**. As a result, data showed all *bla*_NDM-1_-harboring plasmids belong to IncN-typed replicon. The size ranges from 49,215-bp to 63,046-bp and is found in different species, including *K. pneumoniae*, *Escherichia coli* and *Enterobacter cloacae*. The size was smaller than that of the pKPSY-2 plasmid and the replicon varied. Plasmids comparisons showed the plasmid backbone is completely different and only the *bla*_NDM-1_-related region is similar (**Figure 4A**).

**Table 4:** Similar plasmids information in the public database.

Plasmid	Accession number	Identity	Size	Species	Strains	Host	Source	Location	Year	Replicon	Resistance genes
pLK78	KJ440075.1	0.995	56,072	*K. pneumoniae*	CGMHLK78	–	–	China, Taiwan	–	IncN	*ARR-3*, *aac(6’)-Ib-cr*, *aadA1*, *aadA16*, *bla*_NDM-1_, *bla*_OXA-10_, *dfrA27*, *sul1*, *tet*(A)
pLK75	KJ440076.1	0.995	56,489	*Escherichia coli*	CGMHLK75	–	–	China, Taiwan	–	IncN	*ARR-3*, *aac(6’)-Ib-cr*, *aadA1*, *aadA16*, *bla*_NDM-1_, *bla*_OXA-10_, *dfrA27*, *sul1*, *tet*(A)
p4	NZ_CP026590.1	0.994	49,215	*K. pneumoniae*	NUHL30457	Homo sapiens	–	China	2016	IncN	*bla*_NDM-1_, *dfrA14*, *qnrS1*
pEcNDM1	NC_023909.1	0.993	58,228	*Escherichia coli*	EcNDM1	Homo sapiens	–	China	–	IncN	*ARR-3*, *aac(6’)-Ib-cr*, *bla*_NDM-1_, *bla*_OXA-1_, *catB3*, *qnrA7*, *sul1*, *tet*(A)
pC2414-3-NDM	NZ_CP039821.1	0.991	63,046	*K. pneumoniae*	C2414	Homo sapiens	Urine	China	2017	IncN	*bla*_NDM-1_, *dfrA14*, *qnrS1*
pNDM-BTR	KF534788.2	0.990	59,400	*Escherichia coli*	–	–	–	China	–	IncN	*bla*_NDM-1_, *dfrA14*, *qnrS1*
pNDM1-CBG	NZ_CP046118.1	0.990	62,663	*Enterobacter cloacae*	CBG15936	Homo sapiens	Sputum	China	2017	IncN	*bla*_NDM-1_, *dfrA14*, *qnrS1*
pECAZ159_2	NZ_CP019006.1	0.996	47,531	*Escherichia coli*	Ecol_AZ159	Homo sapiens	–	Colombia	2013	IncN	*aac(6’)-Ib-cr*, *aadA16*, *bla*_KPC-2_, *sul1*
pEC881_KPC	NZ_CP019026.1	0.994	59,373	*Escherichia coli*	Ecol_881	Homo sapiens	–	Colombia	2013	IncN	*aac(6’)-Ib-cr*, *aadA16*, *bla*_KPC-2_, *sul1*, *qnrB6*
pEC224_KPC	NZ_CP018945.1	0.993	55,436	*Escherichia coli*	Ecol_224	Homo sapiens	–	Viet Nam	2010	IncN	*ARR-3*, *aac(6’)-Ib-cr*, *aadA16*, *bla*_KPC-2_, *dfrA27*, *sul1*
pKPC-79f0	NZ_CP026169.1	0.993	58,627	*Leclercia* sp.	LSNIH1	–	–	USA	2016	IncN	*bla*_KPC-2_, *dfrA14*
pEC422_KPC	NZ_CP018959.1	0.993	51,885	*Escherichia coli*	Ecol_422	Homo sapiens	–	Ecuador	2011	IncN	*aac(6’)-Ib-cr*, *bla*_KPC-2_, *bla*_OXA-1_, *catB3*, *sul1*
pKPC-56a	CP009867.1	0.992	54,000	*Pantoea* sp.	PSNIH2	–	Hand rail	USA	2013	IncN	*bla*_KPC-2_, *dfrA14*
pKPC-47e	NZ_CP008901.1	0.992	50,333	*Enterobacter hormaechei*	ECNIH3	Homo sapiens	Tacheal aspirate	USA	2011	IncN	*bla*_KPC-2_, *dfrA14*
pKPC-1c5	NZ_CP009881.1	0.992	50,272	*Pantoea* sp.	PSNIH1	–	Shelf	USA	2013	IncN	*bla*_KPC-2_, *dfrA14*
pKPC-3714	NZ_CP026389.1	0.992	50,333	*Leclercia* sp.	LSNIH3	–	–	USA	2016	IncN	*bla*_KPC-2_, *dfrA14*

Tracking of the *bla*_KPC-2_ plasmids identified them in different species, including *E. coli*, *Leclercia* sp., *Pantoea* sp., and *E. hormaechei*. The size was similar to that of the pKPSY-3 plasmid identified in this study, and the replicon was identical (IncN). However, *bla*_KPC-2_-positive plasmids have been found in various countries such as Colombia, Vietnam, Ecuador, and the USA, suggesting that transmission occurred worldwide. Plasmids comparisons revealed that *bla*_KPC-2_-positive plasmids possessed huge similarity (**Figure 4B**). However, the genetic environment of *bla*_KPC-2_ is various among them (**Figure 4B**).

### Comparative genomics analysis of global ST852 clone

**Figure 5:**
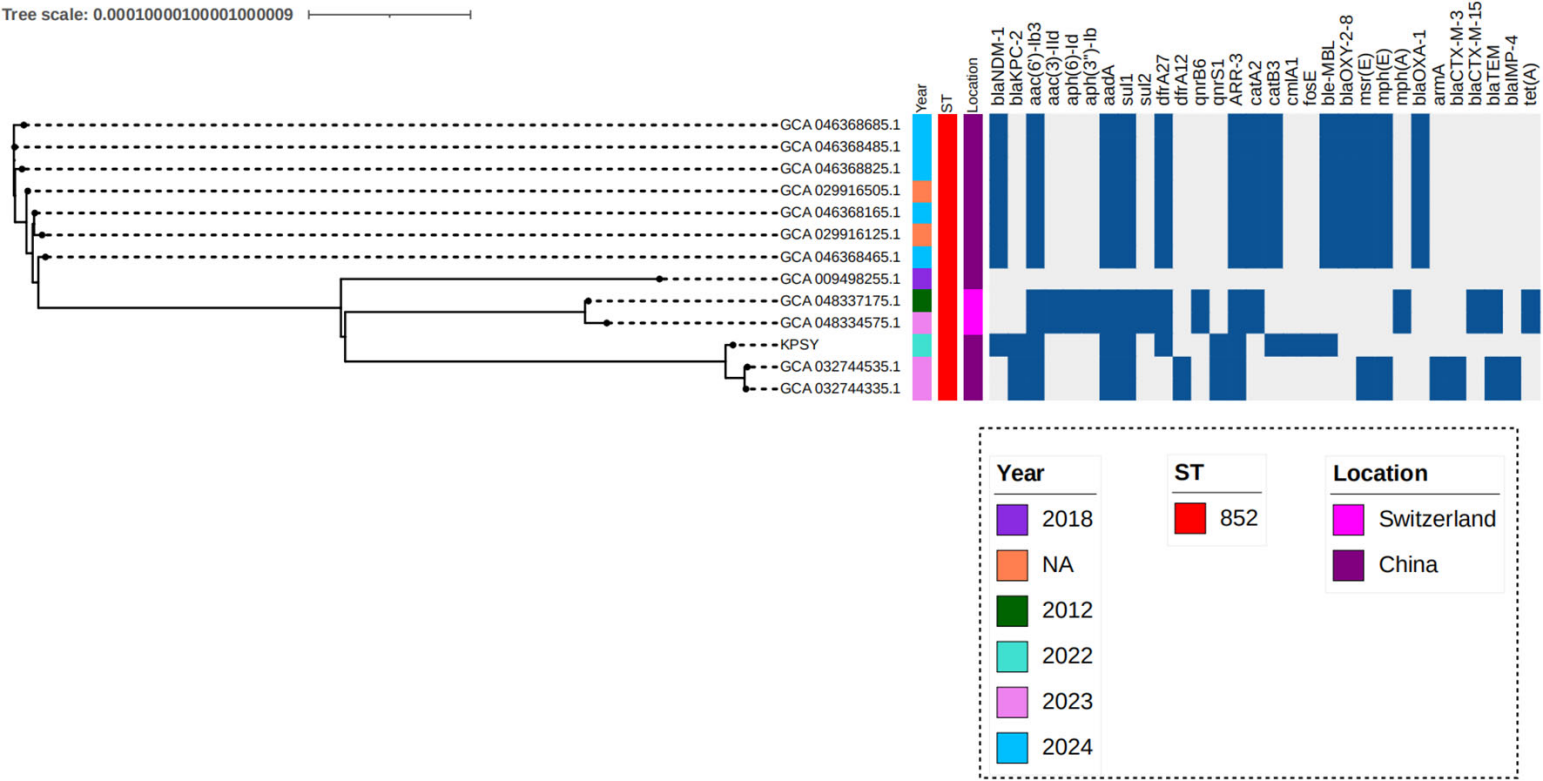
Phylogenetic analysis of ST852 *K. quasipneumoniae* strains. Phylogenetic analysis was conducted the tree was visualized with iTOL v7. Isolates name, location, collection date, ST type and resistance genes are shown.

To analyze the genetic characteristics of the ST852 clone from a global view, comparative genomics analysis of 13 global *K. quasipneumoniae* strains was performed. Data showed 13 ST852 strains were mainly isolated from China (84.62%, 11/13), and the remaining isolates were collected from Switzerland (**Figure 5**). Carbapenemase genes analysis showed 7 strains only harbored *bla*_NDM-1_ gene and two isolates carried *bla*_KPC-2_ and *bla*_IMP-4_, simultaneously. Co-carriage of *bla*_NDM-1_ and *bla*_KPC-2_ has so far only been seen in our strain in China in 2022 (**Figure 5**). In addition, a relatively close genetic relationship with isolates recovered from China in 2023 was observed (**Figure 5**).

## Discussion

Nosocomial outbreaks caused by carbapenem-resistant Enterobacteriaceae (CRE) strains are rapidly emerging worldwide and are a cause of concern (Ernst et al., 2020). Among the clinical strains of CRE, *K. quasipneumoniae* is considered an important nosocomial pathogen that has not been comprehensively studied. The emergence of XDR *K. quasipneumoniae*, specifically carbapenem-resistant isolates, presents a significant challenge for clinical treatment (Ma et al., 2024).

Mobile genetic elements (MGEs), including ISs, integrons (Ins), and transposons (Tns), play particularly important roles in the transfer of resistance genes among the same or different species (Gorbunova et al., 2021). Dong et al. characterized *bla*_KPC-2_- and *bla*_IMP-4_-carrying conjugative IncHI5 hybrid plasmids in a clinical ST852-KL18 *K. quasipneumoniae* strain (Dong et al., 2023). Based on our strain data, we found that the ST and KL types were identical. However, the plasmids (*bla*_KPC-2_- and *bla*_NDM-1_-carrying plasmids) were different, including the plasmid size (> 200 Kb and 58 Kb), plasmid structure (with and without *oriT*, T4SS region, T4CP, relaxase) and the resistance genes context (flanked by IS*Kpn27*-IS*Kpn6* for *bla*_KPC-2_ and no ISs were found in the upstream and downstream for *bla*_KPC-2_). Thus, these data suggested that diverse resistance plasmids were existed in *K. quasipneumoniae*. One interesting finding in this study is that *bla*_KPC-2_-positive plasmids were observed in different species in different countries, suggesting that the IncU-typed plasmid has likely spread globally. Indeed, we found *bla*_KPC-2_-positive pKPSY3 plasmid is a transferable plasmid containing *oriT*, T4SS, T4CP, and relaxase-related genes, further emphasizing its importance in the dissemination of the carbapenem resistance gene.

Moreover, genetic environment analysis of many gene cassettes revealed a complex class 1 integron in *bla*_KPC-2_ and *bla*_NDM-1_-carrying plasmids in the present study. Integrons can capture gene cassettes via *attl* and *attC*, which can contribute to the resistance to different antimicrobial agents and lead to a global resistance crisis (Ghaly et al., 2021). Many studies have shown that IS*26* plays an important role in the spread of resistance genes in gram-negative bacteria (Harmer et al., 2014; Hua et al., 2020). A crucial feature of the IS*26* genetic element is its ability to cointegrate molecules comprising distinct DNA segments (Hua et al., 2020). Here, we found two copies of IS*26* in the large pKPSY2 plasmid flanked by various 8-bp target site duplications (TSD) sequences. When IS*26* relocates, a short sequence (8-bp) at the site they move to (the target site) is duplicated and this appears as one 8-bp direct duplication, named TSD, flanking the IS*26* in its new location (Harmer and Hall, 2024). The same 8-bp sequences could be used as an insertion marker. However, considering the 8-bp different TSD sequences in this study, we thought we are failed to find the direct evidence to indicate this large pKPSY-2 plasmid was formed by cointegration.

It is well-documented that the OCL and KL gene clusters in bacteria are responsible for the biosynthesis of the outer core of lipooligosaccharides and the capsule (Kenyon and Hall, 2022). These are potentially useful epidemiological markers that play key roles in vaccine development (Wyres et al., 2020). In the current study, KL18 had a high identity but was only reported in one strain coharboring *bla*_KPC-2_ and *bla*_IMP-4_ in China. However, this clone has been suggested to be rare.

However, this study has some limitations, such as the need to assess the virulence level of this strain using insect larval or murine models. In addition, the transfer frequency of the *bla*_KPC-2_-positive plasmid was measured by conjugation. Finally, the fitness cost of transconjugants containing the *bla*_KPC-2_ plasmid was tested using the growth curve method.

## Conclusion

In summary, this is the first study to report the complete genome characteristics of the rare KL18-O3/O3a ST852 *K. quasipneumoniae* strain co-carrying *bla*_NDM-1_ and *bla*_KPC-2_. Furthermore, it promoted carbapenem resistance via conjugation. Tracking of *bla*_KPC-2_-harboring plasmids identified them in different species in different countries, suggesting the possible spread of this IncU-type plasmid worldwide. Therefore, XDR *K. quasipneumoniae* and its different resistance plasmids are a public health concern and require further surveillance to avoid their extensive spread in healthcare settings.

## Data Availability

The datasets presented in this study can be found in online repositories. The names of the repository/repositories and accession number(s) can be found below: The genomes of K. quasipneumoniae KPSY strain have been deposited in GenBank under the BioProject: PRJNA1202470.
